# Designing and psychometric properties of the hospitalized patients’ spiritual needs questionnaire (HPSNQ) in the medical-surgical hospital setting

**DOI:** 10.1186/s12904-023-01213-5

**Published:** 2023-08-05

**Authors:** Fahimeh Alsadat Hosseini, Marzieh Momennasab, Joan Guàrdia-Olmos, Shahrzad Yektatalab, Maryam Shaygan, Armin Zareiyan

**Affiliations:** 1 Community Based Psychiatric Care Research Center, School of Nursing and Midwifery, Shiraz University of Medical Sciences, Shiraz, Iran; 2 Department of Nursing, School of Nursing and Midwifery, Shiraz University of Medical Sciences, Shiraz, Iran; 3 Department of Methodology of Behavioral Sciences, Faculty of Psychology, University of Barcelona, Barcelona, Spain; 4 Public Health Department, Health in Disaster & Emergencies Department, Nursing Faculty, Aja University of Medical Sciences, Tehran, Iran

**Keywords:** Validity, Reliability, Questionnaire, Spiritual needs, Hospitalized patients

## Abstract

**Background:**

The evaluation of spiritual requirements in patients can facilitate the delivery of spiritual care as an essential element of holistic healthcare. The objective of this research was to develop and evaluate the psychometric properties of a questionnaire on patients’ spiritual needs in medical-surgical hospital settings.

**Methods:**

This research utilized an exploratory sequential design, involving the creation of a pool of items through both inductive and deductive methods. The questionnaire’s psychometric properties were then assessed using various techniques, such as face and content validity, item analysis, construct validity, internal consistency, stability, confirmatory factor analysis, and the Gradual Response Model of Samejima. The data analysis was conducted using MPLUS software, version 5.1.

**Results:**

The study’s results showed that a four-factor structure (interpersonal connectedness, relationship with God, transcendence, and peaceful environment) with 43 items was successfully extracted through exploratory factor analysis. The confirmatory factor analysis supported the findings of the exploratory factor analysis. The Cronbach’s alpha coefficients for the scale and factors ranged between 0.83 and 0.95. Furthermore, the interclass correlation coefficients for the scale and factors were between 0.89 and 0.96.

**Conclusions:**

The questionnaire designed in this study is a reliable and valid instrument that can be utilized by healthcare, educational, and research institutions to evaluate the spiritual needs of patients in medical-surgical hospital settings.

**Supplementary Information:**

The online version contains supplementary material available at 10.1186/s12904-023-01213-5.

## Background

Concepts like “spirit” and “spirituality” can have different definitions [1]. Everyone’s definition of spirituality is unique and is affected by their own personal beliefs and value systems [2]. According to Joseph et al. (2017), spirituality is a vast, unstructured, natural phenomenon in which one seeks a relationship with a more powerful force or purpose [3]. The concept of spirituality is often considered a broad and inclusive term that can encompass a diverse range of interpretations and meanings [4]. In the realm of nursing, definitions of spirituality often incorporate various components, such as the existence of a higher power, relationships and transcendence, the significance and purpose of life, and the experience of being connected to others [5, 6]. Regardless of the various definitions or conceptualizations, spirituality contributes to the health and wellbeing of people [7, 8].

Incorporating the spiritual needs of patients into their care is a critical aspect of providing holistic care [9]; Nurses must be prepared to provide spiritual care to patients whenever required, including the administration of a spiritual needs assessment, as spiritual distress can arise at any point in a patient’s journey [10]. Therefore, the spiritual needs of patients play a significant role in nursing assessment, care, and overall patient outcomes. As a result, there is a growing global emphasis on the importance of addressing the spiritual needs of patients [11].

Addressing patients’ spiritual needs can lead to significant positive outcomes in patients, including adaptation to crises, pain, anxiety, and depression [12–14]. Contrary to this, there is evidence that nurses do not always adequately address and assess patients’ spiritual needs [15]. Unmet spiritual needs have a significant effect on patient wellbeing [16]. Negative consequences that can result from unaddressed spiritual needs include decreased perceptions of spiritual peace, lowered levels of quality of life, and an elevated risk of depression [17].

To provide appropriate and competent spiritual care, it is imperative that nurses and healthcare professionals conduct an assessment of their patients’ spiritual needs [18]. To conduct a comprehensive spiritual needs assessment, it is necessary to utilize measurement tools that are well-designed, valid, and reliable [19]. The review of existing literature indicates that numerous studies have been carried out utilizing the Classical Test Theory approach to develop and evaluate the psychometric properties of instruments designed to assess the spiritual needs of patients [18, 20–26]. These studies were conducted on patients with cancer, severe illnesses, people with life-threatening illnesses, older patients, patients with a specific and chronic condition, or people at the end of life [18, 20–25]. In several available instruments in the area of patients’ spiritual needs assessment, the generation of items has been mainly via a literature review (a deductive approach) and without conducting a qualitative study [27–30]. Furthermore, in the area of patients’ spiritual needs assessment, a unique culture-based tool is not available [26] and the spiritual needs mentioned in these questionnaires are somewhat different, which can be due to the dependency of these needs on culture, social and historical background, and religious beliefs [30, 31]. Therefore, the results of these studies cannot be generalized to other situations and societies except by using the psychometric assessments of these tools in target populations. Additionally, it is questioned whether these questionnaires are adequate for addressing all of the needs of patients from diverse backgrounds.

For example, the Spiritual Needs Questionnaire (SpNQ) is one of the most important questionnaires assigned to evaluate spiritual needs in particular patients. The SpNQ as a research instrument has 20 items, and as a diagnostic tool, it has 27 items [32]. It is a suitable questionnaire for use with adults who have threatening and non-threatening chronic diseases. This questionnaire was initially developed in Germany. So far, the SpNQ has been translated, validated, and used in different languages and countries [33]. Moeini et al. (2018) conducted a research study in which they translated and performed a psychometric evaluation of the Persian version of the Spiritual Needs Questionnaire among a sample of elderly individuals with chronic diseases. However, a small sample size [100] of elderly people with chronic diseases may affect the structure of the questionnaire and also restrict the generalizability of the study [34]. In the studies of Hatamipour et al. (2017) and Nejat et al. (2016), the psychometric properties of the SPNQ for cancer patients in Iran were assessed [35, 36]. The findings of these studies revealed that the Persian versions of the SpNQ demonstrated high levels of validity and reliability when utilized to evaluate the spiritual needs of cancer patients in Iran [35, 36].

Generally, it has to be considered that although the validity and reliability of the SpNQ were assessed in Iranian culture, they were done for older adults with chronic diseases and cancer patients. The factorial structure of spiritual needs assessment may vary depending on factors such as sample size, population, cultural and religious background, health status, and the number of items included in the measurement tool employed by the researchers [32, 33]. The Persian version of the SpNQ may not be suitable for evaluating the spiritual needs of patients with health conditions other than those for which it has been validated unless validity testing is conducted specifically for these patient groups. Although the validity and reliability of the Persian version of the SpNQ were evaluated in these studies, it remains uncertain whether the questionnaire is fully responsive to the spiritual needs of patients in Iranian culture. This is particularly important to consider given that over 99% of the Iranian population identifies as Muslim [37]. The perception and significance of individual spiritual needs can vary significantly, as spiritual needs are multifaceted and influenced by one’s cultural and religious background [33]. For example, in the study of Faseleh Jahromi and Eslami Akbar (2020), “expressing beliefs”, and “attention to worship” were two main spiritual needs of Iranian hospitalized patients from the nurses’ viewpoints [38]. The study findings indicated that in Iranian and Muslim contexts, spirituality and religion are not distinct concepts, and all spiritual needs are closely intertwined with religious beliefs [38]. Given the cognitive aspect of spiritual needs during illness, it is crucial to conduct qualitative research to develop culturally appropriate questionnaires for evaluating the spiritual needs of patients with other health conditions.

The recognition of the significance of spiritual elements in human beings has led to an increasing acknowledgement of the responsibility of healthcare professionals, especially nurses, to provide spiritual care. This highlights the importance of incorporating spiritual care into healthcare practices [39]. Evaluating the spiritual needs of patients is crucial for providing appropriate and effective spiritual care [40]. Valid instruments are necessary for identifying spiritual needs and evaluating the efficacy of spiritual interventions [27]. In Iran, there are several developed questionnaires in the areas of spirituality [41], spiritual health [42], spiritual care [43, 44], and spiritual wellbeing [45, 46]. The majority of these questionnaires have not been developed for the patients [41–45]. However, there are only a few validated questionnaires in Iran to assess the patients’ spiritual needs [34, 36, 47]. The majority of existing tools in the area of spiritual needs assessment have been designed and validated for a specific group of patients with cancer, severe or life-threatening illnesses, or people at the end of their life in a variety of societies. However, the number of other patients (without cancer, a severe or life-threatening illness, or not being at the end of their life) hospitalized in medical-surgical hospital units is significant. Also, given the influence of health conditions on patients’ spiritual needs [48, 49], it appears that the spiritual needs of individuals with cancer, severe or life-threatening illnesses, or those nearing the end of life may differ to some extent from those of other patient groups. However, it wasn’t found to be a valid tool designed specifically for the assessment of hospitalized patients’ spiritual needs in medical-surgical units without cancer, a severe or life-threatening illness, or being at the end of their life. Some aspects of spiritual needs have a culture-specific expression [50]. The impact of various religious needs on Iranian people’s spiritual needs is significant [51]. Muslims view spirituality and religion (beliefs and practices) as inseparable and do not distinguish between the two concepts [52]; in fact, the concept of religion is intertwined with spirituality [43]. It should be noted that only Oliveira da Silva’s (2020) study employed item response theory to validate the Portuguese version of the SpNQ for use among patients with HIV [53]. IRT can be an effective approach for developing, assessing, and refining questionnaires, producing precise, valid, and relatively brief instruments that minimize response burden [54]. Therefore, the aim of this investigation was to design and assess the psychometric properties of a questionnaire measuring the spiritual needs of Iranian patients with non-severe health conditions who are hospitalized in medical-surgical units, using both classical test theory (CTT) and item response theory (IRT) psychometric methods.

## Methods

### Study design

The present study employed an exploratory sequential design, a widely used method for developing questionnaires. Initially, the researcher conducted a qualitative analysis of the research topic with the participants and utilized the obtained results as a basis for constructing the questionnaire’s items and subscales. In the subsequent stage of data collection, the researcher validated the instrument in a quantitative manner. This methodological approach ensures a thorough and rigorous investigation of the research topic [55]. To evaluate construct validity, a cross-sectional research design was utilized.

### Participants

The eligibility criteria of this study included hospitalization in medical-surgical units, being conscious and oriented to time, person, and place, having non-severe medical-surgical health conditions (with or without a chronic illness), proficiency in the Persian language, age between 18 and 60 years, ability to provide detailed responses to the questionnaire, absence of AIDS or cancer, absence of severe mental or physical health issues, and not being in the end stages of life. During the quantitative phase of the study, the primary researcher (FAH) visited multiple hospital wards (such as surgery, internal medicine, neurology, rheumatology, etc.) at different times of the day (morning, afternoon, and night) to collect data. Following approval from the supervisors and head nurses, the primary researcher requested that eligible patients who were willing to participate in the study complete the self-administered spiritual needs questionnaire. The ward nurses’ statements were also taken into account when assessing patients’ eligibility based on the inclusion criteria.

### Procedure

#### Phase 1. Qualitative study

In the qualitative study, 16 patients were selected through purposive sampling from different medical-surgical wards at three private and public hospitals. During this stage, data was collected through 16 semi-structured and in-depth interviews with 16 individual patients.

During the interviews, the researcher began with open-ended and general questions, including “How has your illness during hospitalization affected your behaviors, needs, or feelings?” and “What things do you think you need more of since your hospitalization?” Follow-up questions were then asked to gain a deeper understanding of the patient’s responses, such as “Could you give an example of an anecdote that relates to this?” and “What makes you happy during hospitalization?” Probing questions were also employed to clarify responses, such as “Can you describe more?” and “What do you mean?” The interviews were not structured initially and lasted between 45 and 60 min, with patients providing consent to record the interviews. The researcher transcribed the recordings after listening to them repeatedly and arranged another interview after data analysis. The interviews continued until data saturation was reached.

In a qualitative study, the principles of qualitative research and ethical consideration were met. For data analysis, the researchers employed the conventional content analysis method developed by Yan Zhang and Barbara M. Wildemuth (2009) [56]. The main stages of content analysis were specified through selecting the unit of analysis, data organization based on open coding, classification based on the available differences and similarities, data reduction, and theme extraction.

The trustworthiness of the research data was assessed using Guba and Lincoln’s criteria [57]. To further augment the trustworthiness of the data, the prolonged engagement method, negative case analysis, and maximum variety in sampling were employed, and the interviews were reviewed by two participants, experts, and colleagues. In addition, the team approach was used to accurately study the data and ensure the consistency of categories with the participants’ opinions.

Bussing’s (2010, 2021) model of spiritual needs, which comprises four fundamental dimensions, serves as the theoretical foundation for the HPSNQ [32, 58]. The model proposed in this study comprises four primary dimensions that are interrelated: connection, peace, meaning or purpose, and transcendence, which can be associated with underlying categories such as social, emotional, existential, and religious factors. The connection dimension encompasses factors such as love, belonging, alienation, and communication with a partner. The peace dimension includes characteristics such as inner peace, hope, balance, and forgiveness, as well as distress and fear of relapse. The meaning and purpose dimension involves factors such as meaning in life, self-actualization, and role function. Lastly, the transcendence dimension includes spiritual resources, a relationship with God or the sacred, and prayer, among other factors [32, 59]. In this regard, the findings of the qualitative phase of this study were presented in the form of four themes, including “relationship with God,” “interpersonal connectedness,” “peaceful environment,” and “transcendence.”

Based on the data obtained from the qualitative phase of the study and a review of the literature on spirituality, spiritual needs, and related questionnaires, a new questionnaire was designed and implemented during the quantitative phase of the study.

#### Instrument

After the qualitative phase was completed, the collected data was used as a basis for developing items for the questionnaire. Using an inductive approach, the researchers generated a pool of items by incorporating the domains and sub-domains associated with the concept of spiritual needs among hospitalized patients. In addition, the researchers reviewed relevant literature and surveys (a deductive approach).

Using the inductive and deductive methods, 151 items were generated. During this stage, a team of experts in the field of spiritual needs reviewed the generated items, assessed their relevance, acceptability, and comprehensibility, and made necessary revisions to the questionnaire. The team also determined the optimal phrasing for each item. Additionally, interviews were conducted with patients hospitalized in medical-surgical units to identify any questions that they found confusing, irrelevant, difficult, annoying, repetitive, or upsetting. Furthermore, they were encouraged to review the questions carefully and suggest revisions to the phrasing and syntax to ensure they were more consistent with their natural style of speaking. In addition, five Likert response scales were used in the questionnaire, ranging from 1 (not at all) to 5 (very high). Finally, in the joint sessions, the research team reviewed the questionnaire. After careful analysis by the research team, certain items were eliminated, revised, or combined with similar items to enhance their relevance and clarity. As a result, a final item pool comprising 76 items was established at the conclusion of this phase.

#### Phase 2. Quantitative study

The questionnaire’s psychometric properties were assessed in a quantitative research study. The study employed availability sampling to select participants from nine medical-surgical wards across three private and public hospitals located in Shiraz, Iran. Qualitative and quantitative face validity were assessed by receiving the 10 subjects’ views about transparency, fluency, wording of items, and stability of the questionnaire for whatever it was considered to measure. To establish content validity, a panel of 15 professionals with expertise in psychometrics and spirituality provided their perspectives. During the qualitative content validity evaluation process, experts assessed various aspects of the questionnaire, including item importance, grammar, appropriate language usage, item placement, and proper scoring. In addition, the quantitative content validity assessment involved determining the content validity ratio (CVR) and content validity index (CVI). The CVR was estimated by asking experts to indicate whether each item was necessary for assessing a construct within a set of items. Experts were asked to rate each item on a scale of 1 to 3, indicating whether the item was not necessary, useful but not essential, or essential for assessing the construct. The resulting content validity ratio (CVR) ranges from 1 to -1. A higher CVR score indicates that the panel members were more in agreement about the necessity of an item in the questionnaire. The formula used to calculate the CVR is CVR = (Ne - N/2)/(N/2), where Ne is the number of panelists who rated the item as “essential” and N is the total number of panelists. The numeric value of the CVR is compared to the Lawshe table to determine its significance. For instance, in the current study with 15 panelists, an item in the questionnaire is considered acceptable if its CVR value is greater than 0.49 [60].

To determine the CVI, experts were requested to evaluate each item’s relevance and clarity on a scale of 1 to 4, with 3 or 4 indicating high relevance or clarity. The number of items rated as relevant or clear was divided by the total number of experts. An appropriate CVI score for each item was considered to be greater than 79%. If the score was between 70% and 79%, the item needed to be revised, while items with a score less than 70% were omitted [61]. Then, a sample of 30 patients was used to conduct item analysis, which aimed to establish initial reliability and identify the items that influenced reliability. Cronbach’s alpha was calculated for this purpose. The correlation coefficient of the items together is considered in this method. If an item has a correlation coefficient of 0.2–0.3 with at least one other item [62] or if its correlation coefficient with the total score is less than 0.3, it is removed from the questionnaire [63, 64]. In addition, if an item’s correlation coefficient with other items is greater than 0.7, it can be eliminated or merged.

To assess construct validity, factor analysis was conducted in this study. According to Boateng et al. (2018), a minimum sample size of 300–450 individuals is recommended for factor analysis [65]. To achieve the necessary sample size for factor analysis, it is suggested that 5–10 individuals per item be included [66]. The HPSNQ used in this study had 43 items with a 5-point Likert scale response, therefore requiring a total of 301 participants (43 items × 7 individuals per item) to validate the questionnaire.

Explanatory factor analysis (EFA) was performed to assess construct validity using data from 301 participants. For construct validity, the patients completed the questionnaires through self-report. Data collection was carried out within four months in 2018. For EFA, the Kaiser-Meyer-Olkin Index (KMO) and Bartlett’s test of sphericity, the principal component analysis method, and varimax rotation were used. As an orthogonal rotation method, the Varimax minimizes the number of components that have high loadings on each factor. To assess construct validity, patients completed the questionnaires through self-report, and data was collected over a four-month period in 2018. The Kaiser-Meyer-Olkin Index (KMO) and Bartlett’s test of sphericity were used for EFA, along with the principal component analysis method and varimax rotation. Varimax is an orthogonal rotation method that minimizes the number of components with high loadings on each factor. Furthermore, the assumption of this rotation method is that there are no correlations between the components [67]. Therefore, in this study, the rotation method of Varimax is used.

To determine the number of constructs, the researchers utilized initial eigenvalues, scree plot, and parallel analysis. Factors with a special value above one (the Kaiser criterion) were extracted as the main factors. In this study, the criteria for keeping an item in the EFA included a factor loading of 0.4 or above, the absence of cross-loading, having at least three items in one factor, theoretical convergence, and a lack of strong inter-item correlation [67]. Convergent validity measures the level of agreement between multiple indicators of the same construct. To determine convergent validity, the factor loading of the items, Composite Reliability (CR), and Average Variance Extracted (AVE) must be calculated [68]. In this study, CR and AVE were calculated for the extracted factors to evaluate the questionnaire’s convergent validity.

To evaluate the final factor construct model of the questionnaire and assess construct validity, the researchers performed confirmatory factor analysis (CFA) on a second sample of 301 hospitalized patients in the second stage of the study. According to the study of Li (2016), “Diagonally weighted least squares (WLSMV) is specifically designed for categorical observed data (e.g., binary or ordinal) in which neither the normality assumption nor the continuity property is considered plausible”. On the other hand, according to a study [69], WLSMV was more accurate and less biased than MLR in estimating factor loadings under most conditions. Therefore, in this study, means and variance-adjusted weighted least squares (WLSMV) were utilized in Mplus 5.1 for the analysis.

In this study, the researchers evaluated the CFA using several models of fit indices, including the Chi-Square Test of Model Fit, TLI (Tucker-Lewis Index), CFI (Comparative Fit Index), RMSEA (Root Mean Square Error of Approximation), and SRMR (Standardized Root Mean Square Residual). CFI and TLI values above 0.9, and RMSEA and SRMR values below 0.08 were considered acceptable fits. Additionally, concurrent criterion validity was established by determining the correlation between the HPSNQ scores and the scores obtained from the cancer patients’ spiritual needs questionnaire. The cancer patients’ spiritual needs questionnaire, which is a valid and reliable tool in Iranian society, was completed by 80 patients simultaneously with the HPSNQ.

The assumptions of the IRT measurement include unidimensionality, monotonicity, local independence, and item invariance [70]. Unidimensionality means that all of the items on a scale measure the same thing [70]. Based on the monotonicity assumption, the probability of an item’s endorsement continues to increase as the levels of an individual’s trait increase [70]. Local independence assumes that the items of a measure should be independent unless they measure the same underlying trait [71]. Item invariance assumption refers to a phenomenon in which estimated item parameters among various populations are constant [70].

The analysis of the instrument involved using Item Response Theory (IRT) analysis, which included assessing the dimensionality of the items, estimating item parameters, and analyzing the structure of the scale [72]. Before conducting the analyses, the researchers assessed the unidimensionality and concordance of the items in the four subscales [72]. The graded response model (GRM) proposed by Samejima in 1969 [73] was used in this study, and the analyses were conducted using MPLUS (version 5.1) [72]. The HPSNQ response format, which uses a graded response option, was found to be suitable for the IRT model used in this study, which considers items in terms of a series of k minus 1, where k represents the number of response options available [74].

In the GRM analysis, the item threshold (difficulty) and discrimination parameters were estimated. The threshold number was calculated by subtracting 1 from the number of response options [72]. In this study, the questionnaire had five response options, resulting in four thresholds. Item difficulty is a concept used in education to indicate the level of difficulty of a particular item in achieving a 0.5 probability of a correct response, taking into account the respondent’s level of the latent variable (theta). However, in the health field, the concept of “location parameter” may be more closely related to the concept of “item difficulty” [75].

The item discrimination parameter is an indicator of how well questionnaire items differentiate between patients with varying levels of the latent trait being measured. The slope parameter at a specific level of theta represents the item discrimination parameter, which can vary in steepness across different items, as shown in the item characteristic curve (ICC). Items with steeper slopes are better at discriminating between patients than those with less steep slopes.

In addition to estimating the item threshold (difficulty) and discrimination parameters, this study also examined the Item Characteristic Curves (ICCs), which are graphical representations showing the probability of endorsing an item in specific categories as a function of the latent trait of the respondents [76]. Information about the items was then presented through Item Information Curves (IICs), which mathematically reflect how much information can be provided by each ICC. The Test Information Function (TIF) was generated by combining all the IICs, which indicates how well the questionnaire can estimate a person’s location along the latent trait. Information plots can be used to identify psychometric information at different points within the range of a latent trait [77].

The study assessed the reliability of the questionnaire by estimating its internal consistency using Cronbach’s alpha coefficient and test-retest reliability using the interclass correlation coefficient. To evaluate the internal consistency and test-retest reliability of the questionnaire, a sample of 301 hospitalized patients, the same as in the exploratory factor analysis phase, completed the questionnaire for internal consistency, while 34 hospitalized patients completed the questionnaire for test-retest reliability evaluation.

SPSS software (version 19) was used for data analysis in the exploratory factor analysis (EFA), parallel analysis, convergent validity, concurrent criterion validity, and reliability stages. However, MPLUS software (version 5.1) was used for data analysis in the confirmatory factor analysis (CFA) stage and item response theory analysis.

### Ethical considerations

Following approval from the Ethical and Research Committee of Shiraz University of Medical Sciences (No. IR.SUMS.REC.1395.S872) and in adherence to ethical research principles, individuals who fulfilled the inclusion criteria were recruited to participate in the study.

## Results

During the qualitative phase, the researchers conducted individual interviews to elucidate the spiritual needs of hospitalized patients. The concept was found to comprise four dimensions: “relationship with God”, “interpersonal connectedness”, “peaceful environment”, and “transcendence”. Based on the study’s qualitative findings, hospitalized patients require a relationship with God, interpersonal connectedness, and a peaceful environment to achieve transcendence.

Upon completion of the first phase, the researchers developed a hospitalized patients’ spiritual needs questionnaire for the medical-surgical hospital setting based on the definition of the concept and its constituent dimensions. The research team utilized inductive and deductive methods to create a preliminary draft of the questionnaire, which consisted of 151 items. Subsequently, the research team consolidated overlapping items, reducing the final number to 76.

### Psychometric properties

#### Face validity

During the qualitative face validity study, patients were interviewed in person to evaluate the accuracy of the writing, wording, and appearance of the tool items. As a result, twenty items were identified that required some corrections.

In the quantitative phase, the impact score of each questionnaire item was computed on a 5-point Likert scale, with 5 indicating “very important” and 1 indicating “unimportant.“ An impact score of IS ≥ 1.5 was deemed acceptable. Three items did not have an acceptable impact score; therefore, they were deleted.

#### Content validity

During the qualitative assessment of content validity, owing to the similarity of some items to other items, and the overlapping of meaning, 5 items were deleted [7, 25, 52, 70, 74], and items [14, 30], [41, 42], and [58, 59] were also merged together. At this stage, 65 questions remained.

During the quantitative content validity assessment, the researchers calculated the Content Validity Ratio (CVR) and Content Validity Index (CVI) using the following methods:

- Content validity ratio (CVR): During this phase of the study, the results were compared to evaluations provided by 15 experts in the field and the criterion established by the Lawshe table. The Lawshe table sets a minimum content validity ratio of 0.49 based on the number of experts. Any item scoring equal to or above 0.49 was considered appropriate and retained. As a result, 45 items were found to have an appropriate content validity ratio.
- Content validity index (CVI): In this study, all items were retained because their Item-Content Validity Index (I-CVI) was greater than 0.79. Furthermore, the Scale-Content Validity Index/Average (SCVI/Ave), which reflects the overall agreement, was 0.94, indicating excellent agreement among the participants.

#### Item analysis

During this stage of the study, the researchers computed the Cronbach’s alpha coefficient for the entire tool, which was found to be 0.948. The Cronbach’s alpha coefficients for all items ranged from 0.945 to 0.950. Furthermore, the correlation coefficient score of two items (24 and 26) with the total correlation and other items of the questionnaire was less than 0.3; therefore, they were eliminated. The correlation coefficient of five items was between 0.72 and 0.80, and according to the opinion of the research team and the presence of different concepts in these items, the decision was made not to merge the items and further examine the items in the factor analysis stage. Eventually, 43 items were involved in the factor analysis phase. The findings are displayed in Table 1.

**Table 1:** Item-Total Correlations and Cronbach’s Alpha of the HPSNQ (n = 301)

NO	Items/ During my hospitalization, I have had the needs …	Corrected item-total correlation	Cronbach’s alpha if item deleted
1	Developing trust in God to improve my sickness.	0.63	0.946
2	Asking God to forgive my sins.	0.56	0.947
3	Praying to God.	0.68	0.946
4	Performing my religious duties.	0.65	0.946
5	Reading the Quran and religious books.	0.70	0.946
6	Resorting to the Imams and divine ones.	0.67	0.946
7	Asking others to pray for my recovery.	0.55	0.947
8	Providing facilities for consulting about hospitalized patient’s religious challenges.	0.40	0.948
9	Having the treatment team next to me every time I need them.	0.48	0.947
10	Being listened by the treatment team carefully.	0.35	0.948
11	Being understood by the treatment team.	0.70	0.946
12	Being behaved respectfully with me.	0.70	0.946
13	Being behaved lovely by the treatment team.	0.61	0.947
14	Receiving hope through the treatment team.	0.68	0.947
15	Meeting my spiritual needs and interests by the treatment team.	0.50	0.947
16	Contributing to my care-based decision-making by the treatment team.	0.49	0.947
17	Effectively facing my fears and anxieties.	0.65	0.946
18	Being beside to family and friends.	0.52	0.947
19	Talking to family members and relatives.	0.55	0.947
20	Behaving lovely by my family.	0.72	0.946
21	Receiving empathy from my family and relatives.	0.78	0.946
22	Understanding that my family strives to meet all my needs.	0.56	0.947
23	Helping other patients according to my abilities.	0.41	0.948
24	Talking to other patients.	0.17	0.950
25	Covering my body from others.	0.43	0.948
26	Not sharing my personal information with others.	0.21	0.950
27	Thinking about how to improve my conditions, beliefs, and behaviors in life.	0.62	0.947
28	Accepting my current situation.	0.66	0.946
29	Being patient in the face of difficulties and hardships.	0.78	0.945
30	Forgiving myself.	0.71	0.946
31	Forgiving others’ wrong treatment toward me.	0.74	0.946
32	Strengthen hope in myself during problems and illness.	0.79	0.946
33	Being able to do activities making me feel useful.	0.30	0.948
34	Being hospitalized in a calm room and ward without any annoyance and noise.	0.47	0.947
35	Being hospitalized in a pleasant environment (in terms of cleanliness, dress and amenities).	0.43	0.948
36	Having the facilities for performing religious orders.	0.69	0.946
37	Listening to relaxing music.	0.30	0.949
38	Studying my favorite books.	0.53	0.947
39	Going to the hospital natural area.	0.44	0.948
40	Seeing the surrounding natural area from the window of my room while I am hospitalized.	0.30	0.949
41	Smelling healthy and fresh air.	0.41	0.948
42	Being pleased with the divine destiny in the field of my illness and treatment.	0.64	0.946
43	Finding the illness positive aspects.	0.30	0.949
44	Helping me to know more about the value of my life and circumstances.	0.57	0.947
45	To endure the hardships, considering the important goals of my life.	0.60	0.946
	Cronbach’s alpha	0.948	

HPSNQ: Hospitalized Patients’ Spiritual Needs Questionnaire

#### Construct validity

Factor analysis is a commonly used and reliable method for determining construct validity, particularly within the field of psychology.

For the exploratory factor analysis (EFA), a total of 301 hospitalized patients with a mean age of 42.09 (SD = 11.96) years participated in the study. Among the participants, 163 (54.15%) were female, 200 (66.45%) were married, and the average duration of hospitalization was 9.26 ± 6.28. Table 2 provides further details regarding the characteristics of the study participants.

**Table 2:** Sociodemographic Profile of Participants

Variables	Categories	Total (n = 602)	EFA Sample (n = 301)	CFA Sample (n = 301)
Gender F *(%)	Female	336 (55.81)	163 (54.15)	173 (57.48)
	Male	266 (44.19)	138 (45.85)	128 (42.52)
**Marital status F (%)**	Single	160 (26.59)	83 (27.57)	77 (25.5)
	Married	395 (65.61)	200 (66.45)	195 (64.78)
	divorced or widowed	47 (7.80)	18 (5.98)	29 (9.63)
**Education level F (%)**	Illiterate	34 (5.65)	15 (4.98)	19 (6.31)
	Primary school	170 (28.24)	80 (26.58)	90 (29.9)
	Diploma	177 (29.40)	82 (27.24)	95 (31.56)
	Bachelor	135 (22.42)	76 (25.25)	59 (19.6)
	PhD and MSc	86 (14.29)	48 (15.95)	38 (12.63)
**Job F (%)**	Self-employed	224 (37.21)	107 (35.55)	117 (38.87)
	Governmental	96 (15.95)	51 (16.94)	45 (14.95)
	Housewife	182 (30.23)	89 (29.57)	93 (30.90)
	Student	28 (4.65)	17 (5.65)	11 (3.65)
	Unemployed	32 (5.31)	14 (4.65)	18 (5.98)
	Retired	40 (6.65)	23 (7.64)	17 (5.65)
**Hospitalized ward F (%)**	Surgery	80 (13.29)	36 (11.96)	44 (14.62)
	Neurology	71 (11.79)	43 (14.29)	28 (9.30)
	rheumatology	41 (6.81)	27 (8.97)	14 (4.65)
	Nephrology	39 (6.48)	21 (6.98)	18 (5.98)
	Internal	96 (15.95)	46 (15.28)	50 (16.61)
	Gastrointestinal	89 (14.78)	41 (13.62)	48 (15.95)
	Obstetric	45 (7.48)	25 (8.30)	20 (6.64)
	Orthopedic	97 (16.11)	43 (14.29)	54 (17.94)
	Urology	44 (7.31)	19 (6.31)	25 (8.31)
**Being religious from the patients’ perspective F (%)**	Yes	364 (60.46)	172 (57.14)	192 (63.79)
	No	85 (14.12)	47 (15.62)	38 (12.62)
	Somewhat	153 (25.42)	82 (27.24)	71 (23.59)

F (%)*: Frequency (Percent)

To ensure the adequacy of the sample, the Kaiser-Meyer-Olkin (KMO) test was conducted and produced a value of 0.928, which exceeded the minimum acceptable value of > 0.6. Additionally, Bartlett’s test was significant (X2 = 8536.099, P < 0.001). The four extracted factors explained a total variance of 58.35%, as shown in Table 3.

**Table 3:** Pattern Matrix Loading of Exploratory Factor Analysis for Attitudes toward HPSNQ

	Items/ During my hospitalization, I have had the needs …	Factor 1	Factor 2	Factor 3	Factor 4
	Interpersonal connectedness				
9	11. Having the treatment team next to me every time I need them.	0.825			
10	12. Being listened by the treatment team carefully.	0.850			
11	13. Being understood by the treatment team.	0.804			
12	14. Being behaved lovely by the treatment team.	0.688			
13	15. Receiving hope through the treatment team.	0.657			
14	16. Meeting my spiritual needs and interests by the treatment team.	0.673			
15	17. Contributing to my care-based decision-making by the treatment team.	0.607			
17	18. Being behaved respectfully with me.	0.562			
18	19. Being beside to family and friends.	0.675			
19	20. Talking to family members and relatives.	0.735			
20	21. Behaving lovely by my family.	0.775			
21	22. Receiving empathy from my family and relatives.	0.732			
22	23. Understanding that my family strives to meet all my needs.	0.718			
23	24. Helping other patients according to my abilities.	0.618			
24	25. Covering my body from others.	0.549			
	**Relationship with God**				
1	1. Developing trust in God to improve my sickness.		0.798		
2	2. Asking God to forgive my sins.		0.826		
3	3. Praying to God.		0.885		
4	4. Performing my religious duties.		0.881		
5	5. Reading the Quran and religious books.		0.830		
6	6. Resorting to the Imams and divine ones.		0.879		
7	7. Asking others to pray for my recovery.		0.755		
8	8. Providing facilities for consulting about hospitalized patient’s religious challenges.		0.809		
34	9. Having the facilities for performing religious orders.		0.881		
40	10. Being pleased with the divine destiny in the field of my illness and treatment.		0.580		
	**Transcendence**				
16	26. Effectively facing my fears and anxieties.			0.802	
25	27. Thinking about how to improve my conditions, beliefs, and behaviors in life.			0.721	
26	28. Accepting my current situation.			0.801	
27	29. Being patient in the face of difficulties and hardships.			0.739	
28	30. Forgiving myself.			0.764	
29	31. Forgiving others’ wrong treatment toward me.			0.709	
30	32. Strengthen hope in myself during problems and illness.			0.733	
41	33. Finding the illness positive aspects.			0.600	
42	34. Helping me to know more about the value of my life and circumstances.			0.756	
43	35. To endure the hardships, considering the important goals of my life.			0.732	
	**Peaceful environment**				
31	36. Being able to do activities making me feel useful.				0.697
32	37. Being hospitalized in a calm room and ward without any annoyance and noise.				0.640
33	38. Being hospitalized in a pleasant environment (in terms of cleanliness, dress and amenities).				0.746
35	39. Listening to relaxing music.				0.504
36	40. Studying my favorite books.				0.638
37	41. Going to the hospital natural area.				0.642
38	42. Seeing the surrounding natural area from the window of my room while I am hospitalized.				0.760
39	43. Smelling healthy and fresh air.				0.743

**Fig. 1:**
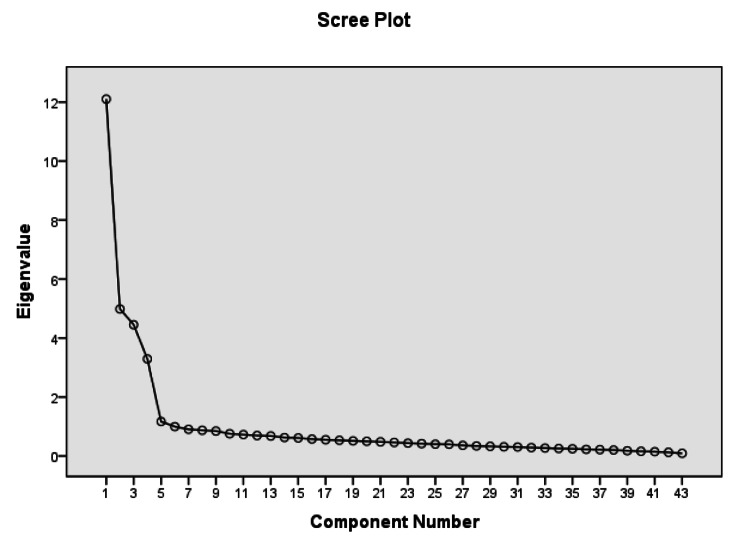
Scree Plot for Determining the Number of Factors in the Questionnaire

The researchers used various methods, including initial eigenvalues, scree plot, and parallel analysis, to determine the number of constructs in the questionnaire. The scree plot showed that five factors explained 60% of the observed variance. However, due to the factor loadings of the two items in the fifth factor being shared with other factors and the factor loadings being lower in this factor compared to other factors, the fifth factor was removed. The parallel analysis method is highly recommended for determining the number of factors. In this method, the eigenvalues obtained from real data are compared with the eigenvalues obtained from randomized data. Factors with eigenvalues greater than those obtained from random data are considered acceptable in this method [67]. In this study, parallel analysis was used to determine the number of factors, resulting in the deletion of the fifth factor (as shown in Table 4). Ultimately, four factors were deemed sufficient to explain the factor structure, accounting for 57.74% of the variance (as seen in Fig. 1).

**Table 4:** Comparative Analysis of Exploratory Factor Analysis using Eigenvalue Criterion and Parallel Analysis using Random Data

Number of factors extracted	Eigenvalues from actual data set	Means Eigenvalues for random data set	95% percentile of random eigenvalues
1	12.000	1,812850	1,898070
2	5.010	1,716852	1,793879
3	4.000	1,646263	1,703515
4	3.000	1,586814	1,642345
5	1.091	1,534328	1,579471

In the subsequent stage, exploratory factor analysis was conducted with a varimax rotation. A minimum acceptable factor loading of 0.4 was set for the degree of correlation between each item and the extracted factors. Items with a high correlation were grouped together accordingly. All items had a factor loading greater than 0.4, and thus all items were retained at this stage.

The composite reliability results for each construct indicated that three factors surpassed the minimum reliability threshold of 0.70, as recommended by Fornell and Larcker (1981) [77]. The criterion for convergent validity was further evaluated by assessing the average variance extracted (AVE) for each factor. However, Table 5 displayed that the AVE for two constructs was less than the recommended threshold of 0.50 [77]. In this study, since the AVE of factors was slightly below 0.50 and the CR of these factors was above 0.7, the domains of the questionnaire and the related items hadn’t changed.

**Table 5:** Factor Composite Reliability (CR) and Average Variance Extracted (AVE)

Factors	AVE	CR
**Factor 1: Relationship with God**	0.668	0.952
**Factor 2: Interpersonal connectedness**	0.494	0.936
**Factor 3: Transcendence**	0.922	0.544
**Factor 4: Peaceful environment**	0.457	0.869

AVE: average variance extracted; CR: composite reliability

The four factors were assigned names based on the content of the items they represented. The first factor (15 agents), the second factor (10 items), the third factor (10 items), and the fourth factor (8 items) were named, respectively, “interpersonal connectedness”, “relationship with God”, “transcendence”, and “peaceful environment”.

A confirmatory factor analysis (CFA) was performed to validate the extracted model using a second sample of 301 hospitalized patients. The participants had a mean age of 45.23 (SD = 10.53), with 173 (57.48%) of them being female, 195 (64.78%) being married, and an average hospitalization duration of 7.46 ± 5.98. Additional participant characteristics are presented in Table 2.

**Fig. 2:**
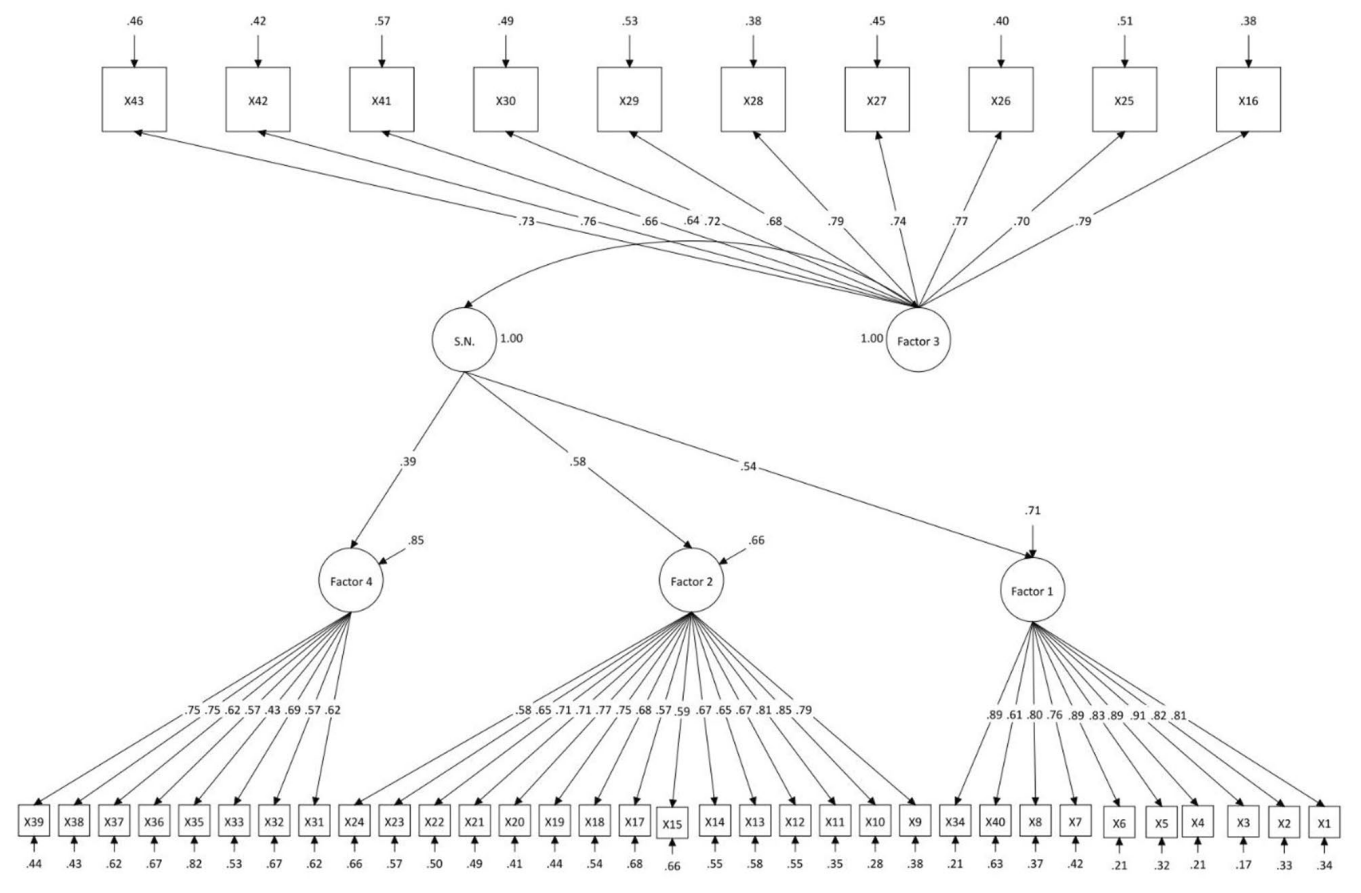
**The second-order CFA model obtained from confirmatory factor analysis** Note: X: Item; Factor 1: Relationship with God; Factor 2: Interpersonal connectedness; Factor 3: Transcendence; Factor 4: Peaceful environment; S.N.: Spiritual needs

The CFA results for the 4-factor structure were as follows: X2 = 1641.82, df = 854, X2/df < 3, CFI and TLI = 0.90, RMSEA = 0.055, SRMR = 0.06, AIC = 24503.29, and BIC = 25003.75. For the second-order CFA, the indices were X2 = 1644.77, df = 856, X2/df < 3, CFI and TLI = 0.90, RMSEA = 0.055, SRMR = 0.06, AIC = 24502.24, and BIC = 24995.29. Based on these results, the second-order CFA model is better than the other one. Figure 2 shows the second-order CFA model. The findings of this study suggest that the model of the questionnaire had a good fit for hospitalized patients in medical-surgical hospital settings, indicating that the theoretical model aligns with the empirical data. This indicates that the four dimensions can effectively reflect the spiritual needs of hospitalized patients in medical-surgical hospital settings.

The concurrent criterion validity was evaluated by comparing the scores of the developed questionnaire with those obtained from a cancer patient’s spiritual needs questionnaire, resulting in an acceptable correlation coefficient of r = 0.76 (p < 0.001).

The evaluation of each item’s parameters, including discrimination and difficulty, was carried out by analyzing the item parameter estimations and dimension patterns of behavior displayed in the ICCs. From the 43 items in the scale, the majority of them demonstrated significant α parameters, except for items 1, 2, and 4 in the peaceful environment dimension. This suggests that most items were effective in discriminating among the response categories, but further attention may be needed to enhance the measurement of the peaceful environment dimension.

The Relationship with God dimension demonstrated high item pattern variability (with a standard deviation greater than 1) and low consistency in item distribution. While all 10 items in this dimension were good discriminators, items 3, 6, and 1 had the highest α parameters (3.287, 2.909, and 2.703, respectively). The remaining items in this dimension also had high discriminating indices (ranging from 1.627 to 2.681), except for item 10, which had lower discriminatory characteristics. Nonetheless, items within the “Relationship with God” dimension demonstrated the highest discrimination parameters, making them the best discriminators of patients with spiritual needs. The test information curve indicated that items within this dimension were generally more informative.

In terms of interpersonal connectedness, items 15, 14, and 4 were found to be the best discriminators based on their α parameters and ICC patterns. Item 7 showed lower discriminatory characteristics, while items 1, 5, 6, 8, 10, and 13 had α parameters less than 1. Within the transcendence domain, the discriminating items ranged from 0.639 to 1.412. Items 8 and 5 were identified as the best discriminators in this dimension, while the discriminating parameters of other items were below 1. Item 1 in this dimension had a lower discriminatory parameter.

According to Table 6, the discriminatory parameters of all items under the peaceful environment domain were below 0.5, which was lower than the parameters of other items in the questionnaire. This implies that only slight discrimination was observed in measuring patients’ spiritual needs. Among the items in this domain, item 6 demonstrated the most effective discriminatory characteristics with a value of 0.426, while item 4 had the least effective ones with a value of 0.045. The Test Information Curve analysis indicated that the items in the peaceful environment dimension were less informative compared to the items in other dimensions.

**Table 6:** Parameter Estimates for the HPSNQ Scale using Item Response Theory (IRT) [72]

Items	α (SE)	β_1_	β_2_	β_3_	β_4_
Relationship with God					
1. Developing trust in God to improve my sickness.	2.703 (0.564)	-6.895	-5.701	-3.108	0.606
2. Asking God to forgive my sins.	2.248 (0.494)	-5.473	-2.992	-0.440	2.186
3. Praying to God.	3.287 (1.027)	-8.039	-5.183	-2.109	1.044
4. Performing my religious duties.	2.681 (0.788)	-3.515	-2.523	-1.020	1.147
5. Reading the Quran and religious books.	2.380 (0.555)	-2.379	-0.947	1.080	3.228
6. Resorting to the Imams and divine ones.	2.909 (0.810)	-6.058	-4.201	-1.804	1.035
7. Asking others to pray for my recovery.	2.038 (0.317)	-4.745	-3.303	-1.063	1.479
8. Providing facilities for consulting about hospitalized patient’s religious challenges.	1.895 (0.432)	-2.398	-1.203	0.261	2.836
9. Having the facilities for performing religious orders.	2.493 (0.673)	-3.479	-2.147	-0.531	2.007
10. Being pleased with the divine destiny in the field of my illness and treatment.	1.627 (0.179)	-6.745	-5.410	-2.266	0.218
**Interpersonal connectedness**					
1. Having the treatment team next to me every time I need them.	0.824 (0.308)	-4.665	-4.438	-3.349	-0.070
2. Being listened by the treatment team carefully.	1.061 (0.366)	-5.747	-5.316	-2.799	0.329
3. Being understood by the treatment team.	1.017 (0.340)	-5.636	-4.903	-2.375	0.680
4. Being behaved lovely by the treatment team.	1.133 (0.284)	-6.472	-5.064	-2.792	0.450
5. Receiving hope through the treatment team.	0.986 (0.311)	-6.333	-5.635	-3.248	0.070
6. Meeting my spiritual needs and interests by the treatment team.	0.933 (0.271)	-5.521	-3.948	-1.660	1.412
7. Contributing to my care-based decision-making by the treatment team.	0.715 (0.235)	-5.265	-4.844	-2.507	0.760
8. Being behaved respectfully with me.	0.879 (0.295)	-6.146	-5.421	-3.592	-0.268
9. Being beside to family and friends.	1.051 (0.261)	-4.944	-4.089	-2.308	0.072
10. Talking to family members and relatives.	0.961 (0.310)	-5.560	-4.395	-2.210	0.636
11. Behaving lovely by my family.	1.042 (0.344)	-5.702	-4.708	-2.912	0.236
12. Receiving empathy from my family and relatives.	1.005 (0.326)	-5.193	-4.484	-2.924	0.463
13. Understanding that my family strives to meet all my needs.	0.918 (0.285)	-5.506	-3.803	-2.001	1.293
14. Helping other patients according to my abilities.	1.377 (0.291)	-5.716	-4.451	-2.010	1.427
15. Covering my body from others.	1.144 (0.276)	-5.253	-4.060	-2.415	0.622
**Transcendence**					
1. Effectively facing my fears and anxieties.	0.639 (0.238)	-5.935	-3.798	-1.786	0.408
2. Thinking about how to improve my conditions, beliefs, and behaviors in life.	0.813 (0.201)	-5.351	-3.412	-1.354	1.452
3. Accepting my current situation.	0.711 (0.252)	-5.291	-3.989	-1.800	0.777
4. Being patient in the face of difficulties and hardships.	0.850 (0.283)	-6.144	-4.677	-2.938	-0.242
5. Forgiving myself.	1.048 (0.280)	-5.199	-3.099	-1.120	1.648
6. Forgiving others’ wrong treatment toward me.	0.745 (0.216)	-5.328	-3.049	-1.055	1.689
7. Strengthen hope in myself during problems and illness.	0.892 (0.252)	-6.194	-5.482	-3.294	0.065
8. Finding the illness positive aspects.	1.412 (0.213)	-4.601	-2.503	-0.529	2.236
9. Helping me to know more about the value of my life and circumstances.	0.796 (0.279)	-5.334	-3.053	-1.189	1.140
10. To endure the hardships, considering the important goals of my life.	0.841 (0.243)	-6.115	-4.261	-2.347	0.350
**Peaceful environment**					
1. Being able to do activities making me feel useful.	0.150 (0.172)	-5.018	-3.188	-1.432	0.587
2. Being hospitalized in a calm room and ward without any annoyance and noise.	0.230 (0.187)	-4.622	-4.330	-2.177	0.105
3. Being hospitalized in a pleasant environment (in terms of cleanliness, dress and amenities).	0.278 (0.166)	-5.742	-5.044	-2.668	0.411
4. Listening to relaxing music.	0.045 (0.177)	-4.599	-2.817	-1.271	1.033
5. Studying my favorite books.	0.307 (0.155)	-3.416	-1.587	-0.325	1.575
6. Going to the hospital natural area.	0.426 (0.167)	-4.689	-2.377	-0.742	1.555
7. Seeing the surrounding natural area from the window of my room while I am hospitalized.	0.384 (0.164)	-4.667	-3.800	-1.746	0.585
8. Smelling healthy and fresh air.	0.412 (0.192)	-5.097	-4.389	-2.803	0.164

Note: α = discrimination parameter; β_1_, β_2_, β_3_ and β_4_ = threshold parameters. The scores in the brackets are the standard error values

**Fig. 3:**
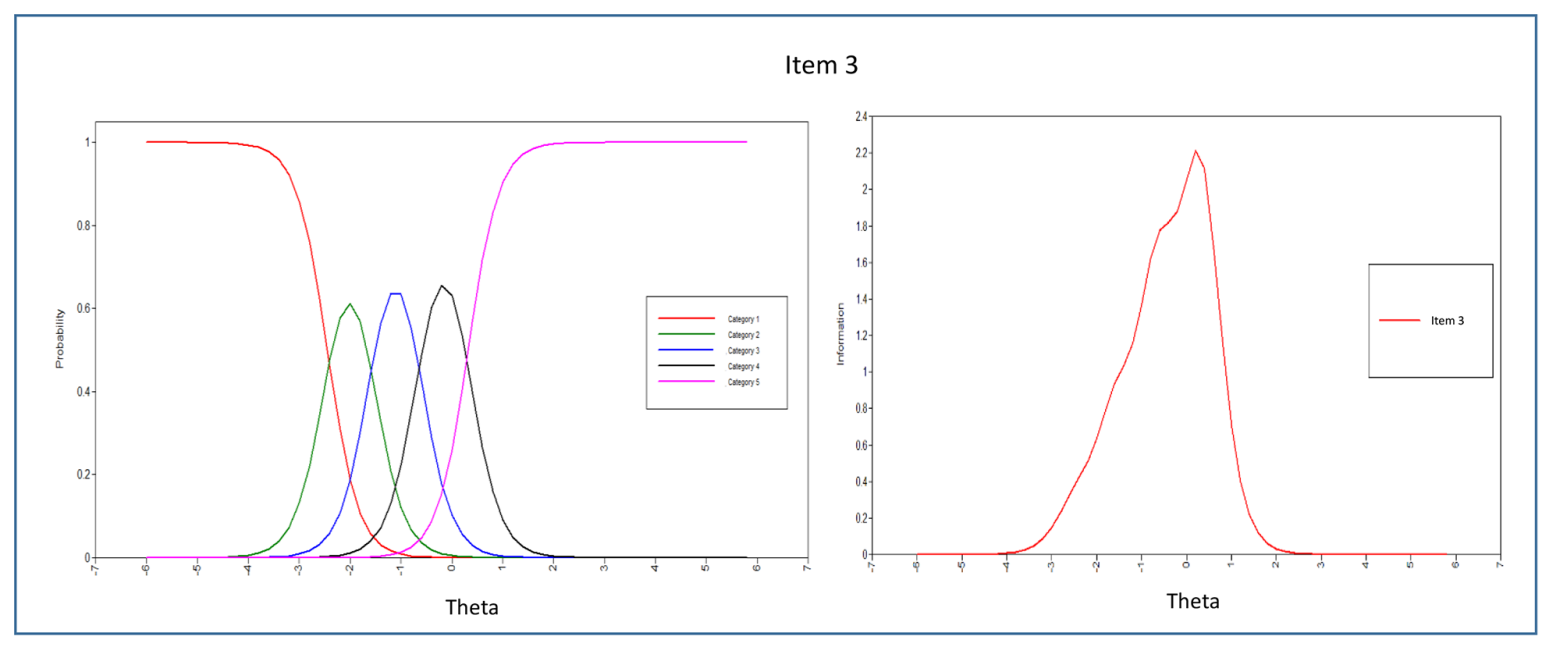
Item Characteristic Curve and Item Information Curve for Item 3

**Fig. 4:**
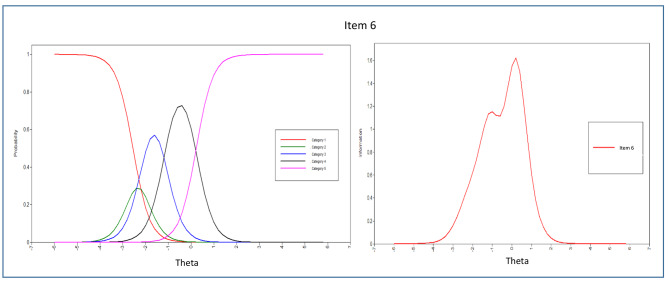
Item Characteristic Curve and Item Information Curve for Item 6

**Fig. 5:**
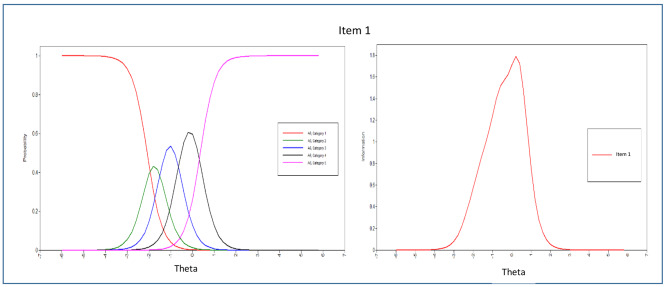
Item Characteristic Curve and Item Information Curve for Item 1

**Fig. 6:**
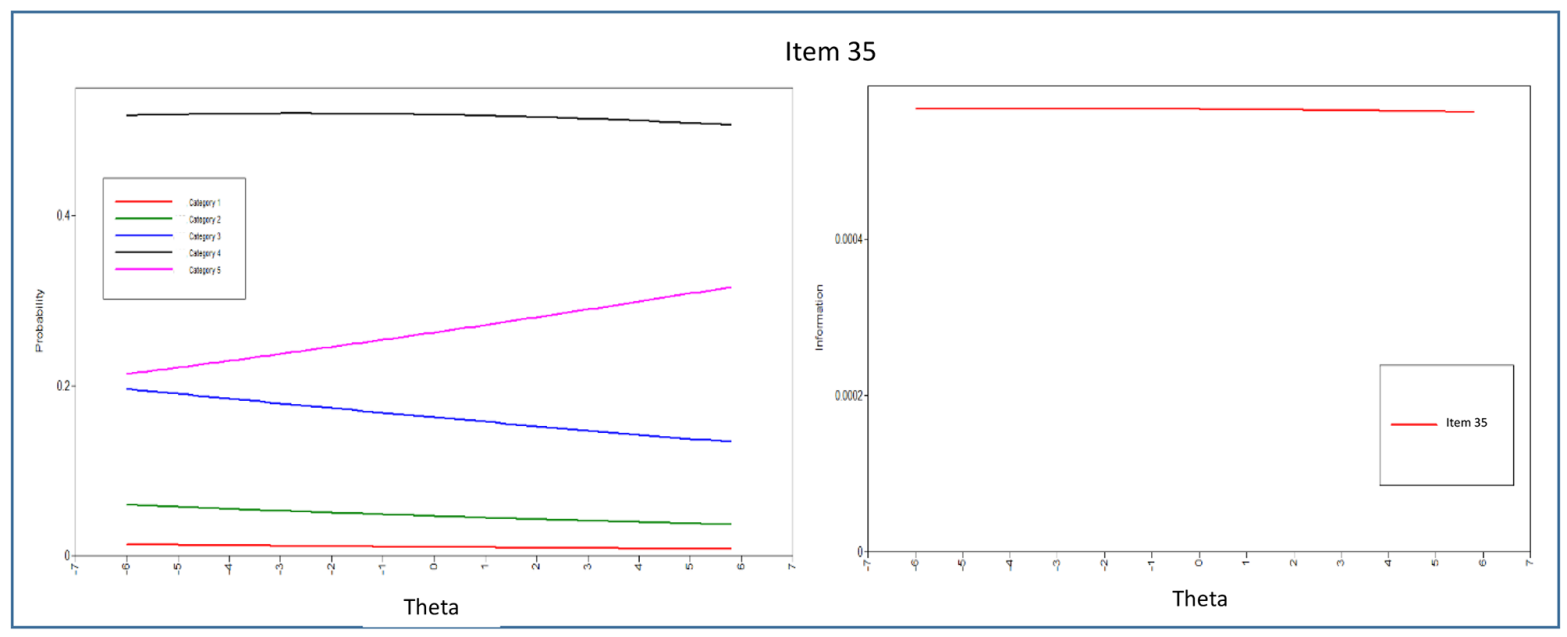
Item Characteristic Curve and Item Information Curve for Item 35

**Fig. 7:**
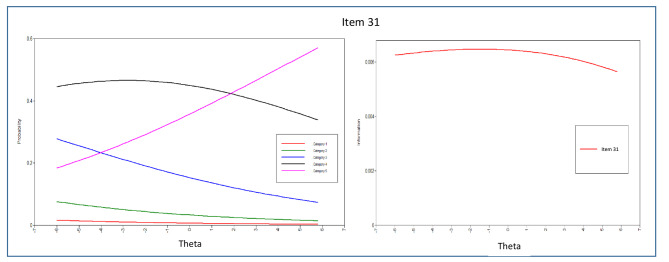
Item Characteristic Curve and Item Information Curve for Item 31

**Fig. 8:**
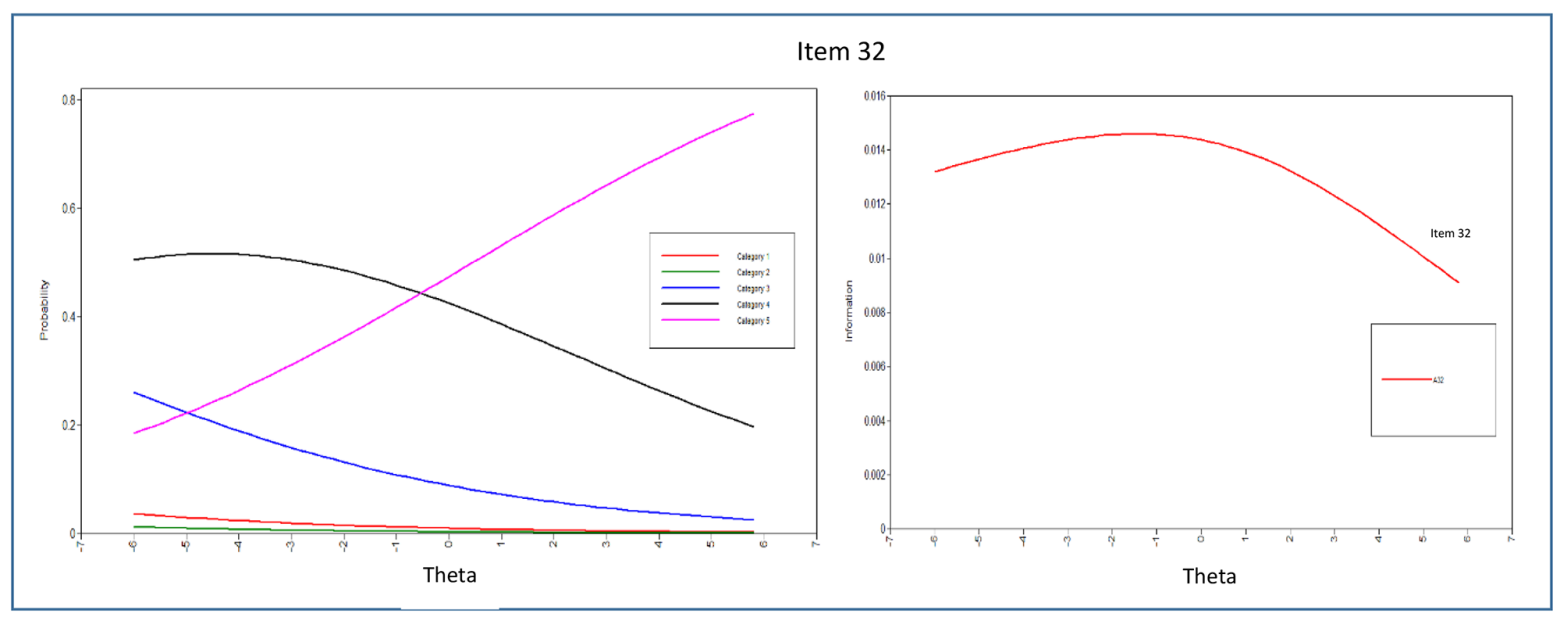
Item Characteristic Curve and Item Information Curve for Item 32

**Fig. 9:**
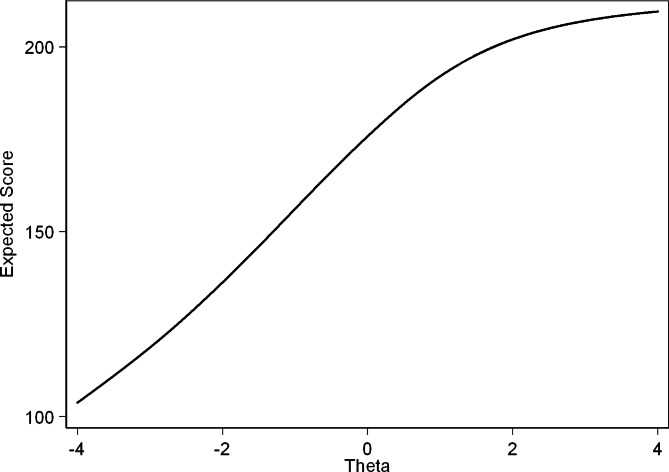
Test Characteristic Curve

The ICCs and IICs of items of the questionnaire with the best and least discriminating characteristics are provided in Figs. 3, 4, 5, 6, 7 and 8. Figure 9 presents the Test Characteristic Curve.

The overall alpha coefficient for the scale was 0.93, while the Cronbach’s alpha for the first, second, third, and fourth factors was 0.93, 0.95, 0.92, and 0.83, respectively. The test-retest reliability results indicated that the total ICC was 0.93, and the ICCs for factors 1–4 were 0.90, 0.96, 0.91, and 0.89, respectively. The final version of HPSNQ can be found in Supplementary File 1.

## Discussion

Considering the dependence of spiritual needs on cultural background and health problems of patients, there is a need to conduct studies in the field of the development and psychometric assessment of spiritual needs questionnaires for hospitalized patients in medical surgical units without cancer, severe or life-threatening illness, or not being at the end of their life. Furthermore, a few studies in the area of the development and psychometric assessment of patient spiritual needs questionnaires have considered the IRT approach in the development and evaluation of these questionnaires. Therefore, this study aimed to design and assess the psychometric characteristics of the spiritual needs questionnaire of patients with non-severe conditions hospitalized in medical-surgical hospital units using both the CTT and IRT psychometric approaches.

In this study, a tool was designed to measure the spiritual needs of patients in hospitals, and its psychometric properties were examined. In the first stage of the study, individual interviews were conducted to understand the meaning of the concept of “spiritual needs” among hospitalized patients in medical-surgical settings. The qualitative findings were categorized into four themes: “relationship with God”, “interpersonal connectedness”, “peaceful environment”, and “transcendence”. Items for the questionnaire were developed based on these themes and sub-themes, as well as a review of relevant literature and questionnaires, using both inductive and deductive approaches. The psychometric properties of the developed questionnaire were then evaluated.

Generating items is a crucial step in developing a questionnaire. In this study, items were generated by utilizing both inductive and deductive approaches. However, some existing instruments for assessing patients’ spiritual needs have mainly relied on a deductive approach of reviewing literature without conducting a qualitative study for item generation [27–30]. It is anticipated that there will be variations in patients’ spiritual needs across different cultures [78]. While some aspects of spiritual needs may be universal, others may be specific to a particular culture, as “spirituality is embedded within culture” [50]. Therefore, there is a continuous effort to develop valid tools that are specific to cultural settings [43, 79, 80]. Based on the findings of this study, although the spiritual needs of Iranian Muslim patients share similarities with those of patients from other cultural backgrounds, there are notable and distinctive spiritual needs specific to Iranian patients, particularly in the religious domain. It is worth mentioning that the majority of patients’ spiritual needs relate to the religious domain of spiritual needs and haven’t been considered in other spiritual needs assessment questionnaires. There is no spirituality without religious thoughts, beliefs, and practices in the Islamic context. In this context, religion provides the spiritual path for salvation and a way of life [52]. Therefore, in this study, the inductive approach of incorporating a deep understanding of the relevant experiences of hospitalized patients and then generating items was considered.

Unlike the present questionnaire, the Spiritual Needs Inventory [81] was designed for hospice cancer patients. In addition, the Spiritual Needs Scale [82] and the Spiritual Needs Assessment for Patients (SNAP) [27] have been specifically designed for cancer patients. In Iran, Hatamipour et al. (2018) [35] and Shokouhi et al. (2021) [83] assessed the psychometric properties of the Persian version of the “Spiritual Needs Questionnaire” for cancer patients. Although the validity and reliability of the SpNQ were evaluated in Iranian culture, it was only for elderly people with chronic diseases and cancer patients. The factorial structure of spiritual needs measurement may differ based on factors such as sample size, various health conditions, cultural differences, and religious backgrounds [33]. As a result, the Persian version of the SpNQ cannot be used to assess the spiritual needs of patients with other health problems unless validation for these groups is performed. Furthermore, as some aspects of spiritual needs are universal while others are culture-specific, this should be taken into consideration when developing tools for assessing spiritual needs [50]. As the role of various religious needs is significant in the spiritual needs of Iranian people, it is questioned whether the Persian SPNQ is responsive to the whole needs of patients in Iranian culture or whether some of the most important religious needs of patients are ignored by using this questionnaire. The current study’s participants had several significant spiritual needs, primarily related to the religious domain, that were not addressed by other spiritual needs assessment questionnaires. This finding shows that the development of culturally appropriate questionnaires for patients’ spiritual needs assessment in this study was important.

In this study, the domains of the hospitalized patients’ spiritual needs questionnaire consisted of “relationship with God”, “interpersonal connectedness”, “peaceful environment”, and “transcendence” which were identified based on the findings of the qualitative phase.

Similar to this study, religious needs have been considered in other patients’ spiritual needs assessment questionnaires [27, 29, 84]. For example, in the SPNQ questionnaire, turning to a higher presence, reading religious or spiritual books, participating in a religious ceremony, praying for yourself or someone else, and meeting a religious person are considered as the patients’ spiritual needs [84]. Also, religious needs in the SNAP included visiting the clergy, doing religious rituals, and providing spiritual texts for the patients [27]. Although many of the religious needs in these assessment tools are usable for the hospitalized patients in this study, some of the specific Muslim religious needs have not been taken into account in these questioners, such as “resorting to the imams and divine ones” or “being pleased with the divine destiny in the field of my illness and treatment.“ In the qualitative study of Karimollahi et al. (2007), one of the main spiritual needs of Iranian Muslim patients was religious need. Consistent with the present study, some of the religious needs of the patients were worship (prayer, reading the Quran, praying (doa), etc.), connectedness with the sacred (with god, prophets, and imams), and religious resources. Despite the fact that religious needs are important spiritual needs all over the world, the religious needs of patients from various backgrounds differ [85]. Therefore, it is crucial to examine and assess the spiritual needs of patients based on the unique cultural and religious background of their society.

In the current study, the domain of “interpersonal connectedness” refers to the patients’ needs for the presence and attention of others, to receive and provide support for others, and to be treated with dignity. Connectedness with others is mentioned as a component of spirituality [2, 86]. A sense of strong and positive interpersonal connectedness is primarily fostered within the context of intimate relationships that involve mutual concern, care, and comprehension [87]. Many of the spiritual needs related to the “connectedness with others” domain that were identified in this study have been addressed in other spiritual needs questionnaires, albeit under different domain names [18, 27, 29, 84]. For instance, in the SpNQ, providing comfort and sharing one’s personal experiences were considered spiritual needs falling within the giving/generativity domain. Also, interacting with others and getting support from others were brought up in the existential and inner peace domains of the questionnaire [84]. In addition, based on the study of Wu et al. (2016), having an interpersonal relationship with the patient, supporting patients, respecting patients’ privacy and dignity, listening to the patients, and showing concern for them are located in the “caring and respect” domain of patients’ spiritual care needs [29]. It seems that although other spiritual needs questionnaires consider the majority of spiritual needs to be in the domain of “interpersonal connectedness,“ the hospitalized patients in this study have some more needs in the area of supporting other people or receiving various types of support from their family and treatment team specifically. The spiritual needs that were identified among the patients in this study seem to be influenced by their distinct cultural and religious backgrounds.

The “peaceful environment” domain of the questionnaire in this study revealed the patients’ desire for a calming and enjoyable treatment environment, as well as a connection to nature. In a previous study by Bussing et al. (2010), items related to the “inner peace” domain of the SpNQ included a wish to be in quiet and peaceful places, an appreciation for the beauty of nature, and the search for inner peace [84]. The need to connect with nature and keep inner peace has also been considered in the Spiritual Care Needs Inventory [29]. But “connecting with nature” wasn’t listed as a patient’s spiritual need in some related questionnaires [18, 27]. In none of the studies in the area of designing and validating the spiritual needs questionnaires, providing a pleasant treatment environment for patients has been considered. Providing a peaceful and pleasant treatment environment (from the aspects of space, scenery, and facilities) and providing a possibility for patients to use the merits of nature were identified as the spiritual needs of patients in this study. It seems that meeting these needs increases inner peace and self-integrity in people [88–90]. Therefore, it is important that these needs be considered in the spiritual needs assessment questionnaires.

Another important spiritual need for patients was transcendence. It refers to improving patients’ self-integrity in difficult disease conditions, searching for meaning and purpose in disease and hospitalization, and submission to and satisfaction with their life. Transcendence is identified as an important dimension of spiritual aspects in several literatures [84, 91, 92]. Similar to the findings of this study, in Jadidi et al.‘s study (2022), transcendence referred to the search for meaning (including life satisfaction) and purpose in life, and the need for peace, stability, and balance. Like the present study, Flotman’s (2021) research indicates that self-transcendence can be used as a coping mechanism during difficult times, allowing for a re-evaluation of one’s sense of purpose and leading to three possible shifts: from blaming to working, from reflection to reflexivity, and from self-awareness to other-awareness. This discovery further emphasizes how self-transcendence can facilitate the exploration of the potential advantages of anxiety [93]. In this line, the SpNQ concluded some items related to seeking meaning in life or suffering and some coping mechanisms [84]. The SNAP has items to assess patients’ self-integrity and ability to cope with any suffering in difficult disease conditions and search for meaning and purpose [27]. In this study, although the spiritual needs in this domain are similar to those in other studies, the patients’ needs in the domain of transcendence may have been affected by their religious backgrounds and beliefs.

Compared to the present study, most previous studies provided limited descriptions about the face and content validity [18, 27, 84] of the process of designing and developing spiritual needs assessment tools for patients. For example, in the studies of Yong et al. (2008) [18], Hermann (2006) [81] and Sharma et al. (2012) [27] face and content validity were assessed using a qualitative method only, and limited descriptions were provided in this regard [94]. In Iran, Hatamipour (2018) assessed the face validity of the Spiritual Needs Assessment Scale using written comments from experts and 10 patients. In addition, the content validity index of this scale was above 0.62, and the scale had a content validity ratio greater than 0.80. However, the target group of this questionnaire was patients with cancer, and there were no reports regarding the face validity of a scale using a quantitative method. The current study utilized the content validity ratio to determine which items were essential for measuring the concept under investigation [95]. By utilizing the content validity index in this study, experts’ opinions were used to identify related concepts [96]. The questionnaire’s Kappa score was found to be excellent, indicating strong agreement among raters regarding the relevance of the items.

Item analysis was conducted in this study prior to evaluating construct validity. Results from exploratory factor analysis suggested that the sample size was sufficient to assess construct validity, with Varimax rotation leading to the identification of 4 domains: relationship with God (10 items), interpersonal connectedness (15 items), transcendence (10 items), and peaceful environment (8 items). The four factors extracted from the HPSNQ accounted for 57.74% of the total variance. The results from the parallel analysis confirmed the identification of the four factors in the questionnaire. The results of the convergent validity of factors of HPSNQ using AVE and CR were relatively acceptable.

Although the current study conducted item analysis to identify the items that affect initial reliability, such analysis was not performed in the studies by Büssing et al. (2010) and Hatamipour et al. (2018) [20, 84]. Item analysis was considered in the development process of some spiritual needs questionnaires [18, 27, 97]. For example, in Hermann’s (2006) study, the item-total correlation ranged from 0.07 to 0.65, leading to the removal of seven items [97]. In Yong et al.‘s (2008) study, 11 items were deleted due to poor consistency with other items or redundancy (correlation coefficient > 0.7), as determined by expert review and pilot testing [18]. However, these studies did not provide comprehensive details on their item analysis procedures, such as reporting Cronbach’s alpha or the correlation coefficient scores of items or which items were removed or merged. So, the current study addressed these limitations.

Much like the present study, exploratory factor analysis has been commonly employed in most research studies to evaluate the construct validity of questionnaires that measure spiritual needs [18, 35, 97, 98]. For example, Büssing et al. (2010) performed exploratory factor analysis on a 19-item questionnaire using a sample of 210 patients in Germany who were suffering from chronic pain, cancer, and other chronic conditions. The Kaiser-Mayer-Olkin value was established at 0.91, and the factor analysis identified four factors—religious needs, existential needs, peace needs, and giving/generosity—that collectively accounted for 67% of the variance [84]. The exploratory factor analysis of the spiritual needs scale developed by Hatamipour (2018) showed five factors and 38 items in the scale. In contrast to the current study, Sharma et al. (2012) did not conduct a factor analysis. However, Sharma et al. (2012) evaluated the construct validity of the “Spiritual Needs Assessment for Patients (SNAP),“ a 23-item questionnaire, by comparing the scores of the questionnaire with those of a single-item measure among 47 cancer patients [27]. In general, these questionnaires were designed for a different target population than the current study and were mainly designed and psychometrically tested in a different culture. Furthermore, in contrast to the current study, no parallel analysis and estimation of AVE and CR were performed after EFA to evaluate the designed questionnaires. Parallel analysis is a commonly recommended method for identifying the appropriate number of factors. In addition, the convergent validity of the questionnaire dimensions can be determined by calculating CR and AVE. So, in this study, we considered parallel analysis, CR, and AVE to evaluate the psychometric characteristics of the HPSNQ.

In the current study, CFA was done after EFA and confirmed the suggested model provided in the EFA phase. In the majority of studies, the construct validity of spiritual need questionnaires hasn’t been evaluated using CFA [18, 27, 29, 30, 97]. The construct validity of the questionnaire was only assessed through CFA in the investigation conducted by Bussing et al. (2018). Using CFA, Bussing et al. (2018) conducted a study in Germany involving individuals with chronic illnesses, elderly individuals, and healthy individuals and confirmed the four-factor structure of the SpNQ with 20 items that had been previously established (CFI = 0.96, TLI = 0.95, RMSEA = 0.04, SRMR = 0.03) [99]. Confirmatory factor analysis is typically conducted after establishing the correlation matrix or factor construct and involves testing a theory and hypothesis about the factor construct in question. In this study, various commonly used goodness-of-fit models were examined based on accepted thresholds [100].

To assess concurrent criterion validity in this study, the researchers utilized Hatamipour’s (2018) scale [20]. The findings indicated a satisfactory correlation between the present questionnaire and Hatamipour’s scale. Concurrent criterion validity has not been tested in some psychometric studies on patients’ spiritual needs [27, 98], in contrast to the current study. However, Hermann (2006) found a weak negative correlation (-0.17) between the number of unfulfilled spiritual needs and life satisfaction, as measured by the Cantril ladder [97]. In addition, these questionnaires were designed for dying patients in the USA. In addition, in the study of Hatamipour (2018), concurrent criterion validity was carried out by estimating the correlation coefficient between the scores obtained from the scale and the “spiritual needs questionnaire” provided by Bussing et al. (r = 0.74 and p < 0.001). Although the context in which the authors of this study assessed the psychometric properties of the scale is similar to the context of this study, the scale was used to assess the spiritual needs of patients with cancer, and there is a probability that it does not cover the whole religious and spiritual needs of patients. In the current study, the concurrent criterion validity of the spiritual needs of Iranian hospitalized patients in medical-surgical hospital settings was estimated to be acceptable (r = 0.76).

To evaluate reliability in this study, internal consistency and stability (test-retest method) were utilized over a two-week interval. The results indicated satisfactory Cronbach’s alpha and ICC values for both the total scale and its factors.

While internal consistency through Cronbach’s alpha has been the primary tool used to assess the reliability of spiritual needs questionnaires in similar studies, stability has not been assessed in most existing instruments [18, 29, 30, 97, 98]. The strong stability observed in the present questionnaire implies that a respondent’s score will remain consistent over time, which is a characteristic that other questionnaires may not possess.

A noteworthy aspect of the current investigation is the use of a graded response model of IRT to create a spiritual needs questionnaire for hospitalized patients. IRT is conceptually more rigorous than CTT. The parameters of an item do not depend on the subject samples, but the plotting of the item’s characteristics is more profound and reasonable than CTT [101]. IRT can be used to improve test quality, although it does not completely replace CTT. For example, in contrast to the CTT, which assumes a uniform standard error for all levels of the trait, the IRT allows for the computation of the measurement’s standard error for each level of the scale, each item, and each respondent [102].

Although IRT has demonstrated its utility in the field of psychometric research, it has only been applied to evaluate the psychometric characteristics of the Spiritual Needs Questionnaire (SpNQ) in its Portuguese version for HIV + patients in Oliveira da Silva’s (2020) study. The study showed that the SpNQ items were appropriately discriminating and had varying levels of difficulty, indicating that the questionnaire has good psychometric properties [53]. This suggests that IRT could and should be utilized to enhance the quality of assessment questionnaires related to patients’ spiritual needs [103].

By utilizing Samejima’s Graded Response Model (SGRM) of IRT, the present study was able to conduct a more comprehensive evaluation of the strengths and weaknesses of the items comprising the HPSNQ, including an assessment of each item’s discrimination and difficulty, as well as the patterns of their characteristic curves. The IRT analysis conducted in this study revealed that the majority of items had acceptable levels of discrimination, while the dimensions of “interpersonal connectedness” and “transcendence” had moderate, but not very high or low, discrimination. All items under the “relationship with God” dimension demonstrated very high levels of discrimination, making them more informative, while the items under “peaceful environment” had relatively low discriminatory characteristics. This may be due to the extreme nature of the response to the “peaceful environment” item or the social and cultural conditions of patients and medical facilities in governmental hospitals, which may affect responses to items with low discriminatory characteristics. Future studies could explore whether rephrasing or eliminating these items in future versions would be more appropriate.

In general, this investigation found that while the HPSNQ exhibited satisfactory psychometric properties from an IRT perspective, there are still indications for improvement. The under-examined construct appears to have a considerable influence on item discrimination and item information value. The HPSNQ dimensions seem to be constructs with only one end or quasi-continuous traits, or they may be latent types with or without the trait, which requires further research. Nevertheless, refining the wording or eliminating certain items or response options in the questionnaire may substantially enhance its reliability.

This study had limitations, such as the failure to consider the disease prognosis of hospitalized patients, which may have an influence on their spiritual needs. Moreover, the participating patients were Muslim and had an Iranian cultural and social context; therefore, the current scale cannot be used without assessing the psychometrics in other societies. This study excluded patients with AIDS or cancer, acute mental or physical disorders, or those in the end stages of life due to the nature of their health conditions and their potential impact on their spiritual needs. Therefore, it is recommended that further investigations be conducted to explore the spiritual needs of these vulnerable subgroups of hospitalized patients in various contexts within medical-surgical hospital settings.

## Conclusions

This study found that the HPSNQ questionnaire is a reliable and valid tool for evaluating the spiritual needs of patients admitted to medical-surgical Iranian settings. However, a comprehensive analysis of each necessary characteristic and related domain revealed that while the discrimination patterns of most items were satisfactory, certain items (such as those in the “peaceful environment” domain) may need to be revised or eliminated. The nature of the construct being examined appears to have a significant influence on item discrimination and information value.

This questionnaire can be used by the healthcare team, researchers, educators, and policymakers to assess the patients’ spiritual needs, which ultimately leads to providing spiritual care. It is suggested that more psychometric studies be conducted on the questionnaire in other societies and cultures.

### Electronic supplementary material

Below is the link to the electronic supplementary material.

Supplementary Material 1

## Data Availability

The datasets resulting from the current study, which were generated and/or analyzed, cannot be made public due to patient confidentiality. Nevertheless, the corresponding author can provide access to these datasets upon reasonable request.

## Declarations

**Ethics approval and consent to participate**.

The Ethics Committee of Shiraz University of Medical Sciences approved the study (IR.SUMS.REC.1395.S872), which adhered to the Declaration of Helsinki and all applicable guidelines and regulations. Written informed consent was obtained from all participants. The methods employed were conducted in accordance with relevant regulations and guidelines.

**Consent for publication**.

## List of abbreviations

SPSS: Statistical Package for Social Sciences
EFA: Explanatory Factor Analysis
CFA: Confirmatory Factor Analysis
KMO: Kaiser-Meyer-Olkin Index
AVE: Average Variance Extracted
CR: Composite Reliability
WLSMV: Weighted least squares
TLI: Tucker-Lewis Index
CFI: Comparative Fit Index
RMSEA: Root Mean Square Error of Approximation
SRMR: Standardized Root Mean Square Residual
IRT: Item response theory analysis
GRM: Graded Response Model
ICC: Item Characteristic Curve
IIC: Item Information Curve
TIF: Test Information Function
