# Supplementary material for: Designing and psychometric properties of the hospitalized patients’ spiritual needs questionnaire (HPSNQ) in the medical-surgical hospital setting
Source: BMC Palliat Care. 2023 Aug 5;22:112. doi: 10.1186/s12904-023-01213-5 (PMC10403866; doi:10.1186/s12904-023-01213-5)
Supplement: Supplementary file 1 — Supplementary Material 1 [file 12904_2023_1213_MOESM1_ESM.docx]

**Supplementary File 1.** Standardized Version of the Hospitalized Patients' Spiritual Needs Questionnaire (HPSNQ) after Psychometric Assessment

| **Items/ During my hospitalization, I have had the needs …** | **Not at all**  **1** | **Slightly 2** | **Moderately 3** | **Very 4** | **Very high**  **5** |
| --- | --- | --- | --- | --- | --- |
| **Relationship with God** |  |  |  |  |  |
| 1. Developing trust in God to improve my sickness. |  |  |  |  |  |
| 2. Asking God to forgive my sins. |  |  |  |  |  |
| 3. Praying to God. |  |  |  |  |  |
| 4. Performing my religious duties. |  |  |  |  |  |
| 5. Reading the Quran and religious books. |  |  |  |  |  |
| 6. Resorting to the Imams and divine ones. |  |  |  |  |  |
| 7. Asking others to pray for my recovery. |  |  |  |  |  |
| 8. Providing facilities for consulting about hospitalized patient’s religious challenges. |  |  |  |  |  |
| 9. Having the facilities for performing religious orders. |  |  |  |  |  |
| 10. Being pleased with the divine destiny in the field of my illness and treatment. |  |  |  |  |  |
| **Interpersonal connectedness** |  |  |  |  |  |
| 1. Having the treatment team next to me every time I need them. |  |  |  |  |  |
| 2. Being listened by the treatment team carefully. |  |  |  |  |  |
| 3. Being understood by the treatment team. |  |  |  |  |  |
| 4. Being behaved lovely by the treatment team. |  |  |  |  |  |
| 5. Receiving hope through the treatment team. |  |  |  |  |  |
| 6. Meeting my spiritual needs and interests by the treatment team. |  |  |  |  |  |
| 7. Contributing to my care-based decision-making by the treatment team. |  |  |  |  |  |
| 8. Being behaved respectfully with me. |  |  |  |  |  |
| 9. Being beside to family and friends. |  |  |  |  |  |
| 10. Talking to family members and relatives. |  |  |  |  |  |
| 11. Behaving lovely by my family. |  |  |  |  |  |
| 12. Receiving empathy from my family and relatives. |  |  |  |  |  |
| 13. Understanding that my family strives to meet all my needs. |  |  |  |  |  |
| 14. Helping other patients according to my abilities. |  |  |  |  |  |
| 15. Covering my body from others. |  |  |  |  |  |
| **Transcendence** |  |  |  |  |  |
| 1. Effectively facing my fears and anxieties. |  |  |  |  |  |
| 2. Thinking about how to improve my conditions, beliefs, and behaviors in life. |  |  |  |  |  |
| 3. Accepting my current situation. |  |  |  |  |  |
| 4. Being patient in the face of difficulties and hardships. |  |  |  |  |  |
| 5. Forgiving myself. |  |  |  |  |  |
| 6. Forgiving others’ wrong treatment toward me. |  |  |  |  |  |
| 7. Strengthen hope in myself during problems and illness. |  |  |  |  |  |
| 8. Finding the illness positive aspects. |  |  |  |  |  |
| 9. Helping me to know more about the value of my life and circumstances. |  |  |  |  |  |
| 10. To endure the hardships, considering the important goals of my life. |  |  |  |  |  |
| **Peaceful environment** |  |  |  |  |  |
| 1. Being able to do activities making me feel useful. |  |  |  |  |  |
| 2. Being hospitalized in a calm room and ward without any annoyance and noise. |  |  |  |  |  |
| 3. Being hospitalized in a pleasant environment (in terms of cleanliness, dress and amenities). |  |  |  |  |  |
| 4. Listening to relaxing music. |  |  |  |  |  |
| 5. Studying my favorite books. |  |  |  |  |  |
| 6. Going to the hospital natural area. |  |  |  |  |  |
| 7. Seeing the surrounding natural area from the window of my room while I am hospitalized. |  |  |  |  |  |
| 8. Smelling healthy and fresh air. |  |  |  |  |  |
